# Jute Responses and Tolerance to Abiotic Stress: Mechanisms and Approaches

**DOI:** 10.3390/plants10081595

**Published:** 2021-08-03

**Authors:** Khussboo Rahman, Naznin Ahmed, Md. Rakib Hossain Raihan, Farzana Nowroz, Faria Jannat, Mira Rahman, Mirza Hasanuzzaman

**Affiliations:** Department of Agronomy, Faculty of Agriculture, Sher-e-Bangla Agricultural University, Sher-e-Bangla Nagar, Dhaka 1207, Bangladesh; khussboorahman1305594@sau.edu.bd (K.R.); naznin1305291@sau.edu.bd (N.A.); rakib.raihan1406185@sau.edu.bd (M.R.H.R.); farzana.nowroz@sau.edu.bd (F.N.); fariasnigdha1506589@sau.edu.bd (F.J.); mirarahman73@gmail.com (M.R.)

**Keywords:** abiotic stress, antioxidant defense, bast fiber, climate change, drought, fiber crops, geotextile, oxidative stress, salinity, tossa jute, waterlogging, white jute

## Abstract

Jute (*Corchorus* spp.) belongs to the Malvaceae family, and there are two species of jute, *C. capsularis* and *C. olitorious.* It is the second-largest natural bast fiber in the world according to production, which has diverse uses not only as a fiber but also as multiple industrial materials. Because of climate change, plants experience various stressors such as salt, drought, heat, cold, metal/metalloid toxicity, and flooding. Although jute is particularly adapted to grow in hot and humid climates, it is grown under a wide variety of climatic conditions and is relatively tolerant to some environmental adversities. However, abiotic stress often restricts its growth, yield, and quality significantly. Abiotic stress negatively affects the metabolic activities, growth, physiology, and fiber yield of jute. One of the major consequences of abiotic stress on the jute plant is the generation of reactive oxygen species, which lead to oxidative stress that damages its cellular organelles and biomolecules. However, jute’s responses to abiotic stress mainly depend on the plant’s age and type and duration of stress. Therefore, understanding the abiotic stress responses and the tolerance mechanism would help plant biologists and agronomists in developing climate-smart jute varieties and suitable cultivation packages for adverse environmental conditions. In this review, we summarized the best possible recent literature on the plant abiotic stress factors and their influence on jute plants. We described the possible approaches for stress tolerance mechanisms based on the available literature.

## 1. Introduction

Global climatic change disturbs ecological fitness and induces several abiotic stresses, which are considered as the significant constraints for crop productivity, namely, drought, waterlogging/flooding, salinity, extreme temperature (high and low), toxic metals/metalloids, etc. [1]. Abiotic stress can hamper different morpho-physiological and biochemical features of plants, which leads to a considerable decrease in plant growth and productivity.

Jute is the second most important fiber crop in the world in terms of cultivation and usage [2]. Jute belongs to the Malvaceae family, and *Corchorus capsularis* and *C. olitorious* are the two commercial species of jute. Jute is the longest, cheapest, and one of the strongest vegetable fibers that is extracted from stem bark. In the Indian sub-continent, jute is considered as an important cash crop and has multipurpose usages like food, fibers, fuel, industrial materials, soil enrichment, etc. Jute fiber is 100% biodegradable and eco-friendly [3]. Worldwide, at least 20 countries produce jute, and Bangladesh and West Bengal of India produce approximately 85% of the world’s jute production [4]. In the era of climate change, because of unfavorable climatic conditions, jute yield and quality is gradually decreasing. Temperature and rainfall are the most dominating components for jute production. In recent years, a fluctuation in temperature and rainfall has been seen, and as a result the production of jute is decreasing [5]. Salt stress negatively affects jute growth and physiological parameters, which subsequently reduces yield quality [6]. Drought reduces germination as well as seedling characteristics, which has a caused 20 to 30% loss of fiber yield and decreased fiber quality [7,8]. Though jute is tolerant to waterlogging at the mature stage, at the seedling stage, it become more susceptible towards waterlogging [9,10]. Other stresses like toxic metals/metalloids, heat, and high light also have a negative effect on jute growth and physiology [2,11]. Under stressful conditions, the excess generation of reactive oxygen species (ROS)-induced oxidative damage in jute can be scavenged by a large number of ROS detoxifying antioxidant enzymes [12,13].

To mitigate the negative effect of abiotic stress, as well as to maintain sustainable production, different conceivable approaches can be taken. Agronomic practices like the adjustment of sowing time, nutrient management, organic matter management, tillage, proper irrigation, well-drainage, and the use of tolerant varieties help to ameliorate the negative effects of abiotic stress. The application of different types of biostimulants can be considered a fruitful strategy to increase the tolerance capacity of plants against conditions of stress [14,15]. Recently, several genetic and molecular approaches have been used to develop the stress tolerance of jute. The genome sequence of jute has also been revealed, which created a new avenue for molecular breeding [16].

To increase yields and improve fiber quality, plant biologists should know the actual plant responses under various forms of abiotic stress. Besides, the development and dissemination of stress-tolerant varieties should be introduced to improve jute production. Though abiotic stress substantially hampers growth, physiology, and productivity, a few studies have been conducted on the abiotic stress responses of jute. However, there are hardly any comprehensive reviews that describe the multiple abiotic stress responses and forms of tolerance. This review describes the morpho-physiological responses of jute under abiotic stress and feasible approaches to enhance abiotic stress tolerance.

## 2. Jute Responses to Abiotic Stress

Abiotic stress can hamper different morpho-physiological and biochemical features of jute plants, which leads to a considerable decrease in plant growth and productivity (Table 1). However, the effects of abiotic stress depend on the type of stressor, the duration and extent of stress, the crop species, the growth stage, and other environmental factors.

### 2.1. Morphophysiological Responses of Jute under Different Abiotic Stress

#### 2.1.1. Salinity

Salinity is considered as the vital environmental stress, which significantly reduced germination, plant vigor, and crop yield [40]. About 20% of irrigated land and 6% of world land is covered with salinity [41]. El Moukhtari et al. [42] stated that by creating osmotic stress, ionic imbalance and toxicity salt stress severely affect plant physiology and metabolism.

**Figure 1:**
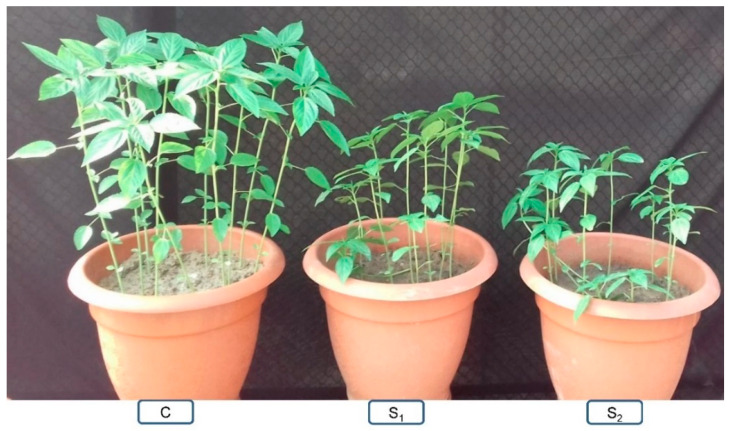
Effect of different levels of salt stress on *C. olitorius* plants; C—control, S_1_—200 mM NaCl, and S_2_—400 mM NaCl.

Jute showed dynamic responses under salt stress. No germination took place above 160 mM when nine varieties of *C. olitorius* were exposed in different doses of NaCl (100, 160, 240, and 300 mM), whereas a decline in the rate of germination at 100 and 160 mM was observed in a dose-dependent manner [20]. Among nine varieties, they recorded maximum reduction of germination (38%) in the S-19 variety and a minimum (18%) in JRO-128 at 160 mM NaCl-induced salt stress over the control. The highest and lowest salt tolerance indices were found in S-19 (77%) and JRO-128 (30%), respectively at 160 mM NaCl, which indicates the susceptibility and tolerance of S-19 and JRO-128 under salt stress. Shoot and root length reduced with the increase in NaCl levels. In another experiment, Naik et al. [6] exposed five *C. capsularis* genotypes in different levels of NaCl (100, 150, 200, and 250 mM NaCl) and the observed highest reduction in shoot length (50%), root length (40%), number of leaves (>70%), and RWC (39%) in the JRC-517 variety at 250 mM NaCl. A contrasting association was also observed between photosynthetic pigments and the salt level, which provoked a reduction in Chl pigments. The highest Pro and protein content were observed in JRC-532, which expressed plants’ salt tolerance characteristics. Similar results were also found in five mutants of *C. olitorius* where growth and yield parameters decreased at 4 and 6 dS m^−1^ salinity [18]. In our recent study, we exposed 15-d-old *C. olitorius* (cv. O-9897) seedlings at different levels (200 and 400 mM NaCl) of salt stress and observed a reduction in growth parameters in both treatments in a dose-dependent manner at 25 days after sowing compared to the control (Figure 1).

Ghosh et al. [19] demonstrated a reduction in shoot length, root length, and RWC of *C. olitorius* (cv. O-9897) by 39, 30, and 9% at 100 mM NaCl and 59, 60, and 21% at 200 mM NaCl, respectively, over the control. However, Chl content (SPAD value) increased by 5% at 100 mM NaCl but reduced by 13% at 200 mM NaCl over the control. Tareq et al. [22] also exposed five varieties of *C. capsularis* at 100 mM and 200 mM NaCl and observed a reduction in growth (plant height and branches plant^−1^) and yield parameters (number of seed capsule^−1^, seed yield plant^−1^, and 1000-seed weight) with the extent of salt stress. They also observed an increase in false seed content with the increased level of salt stress. Yakoub et al. [23] measured that g*_s_*, P*_n_*, and T*_r_* of *C. olitorius* decreased by 86, 75, and 75%, respectively, at 175 mM NaCl over the control. The leaf area and yield parameters, i.e., the number of pods plant^−1^, the lengths of pods (cm), and the number of seeds pod^−1^ were also decreased with the extent of salt stress, and the seed yield reduced by 37% at 175 mM NaCl, corresponding to the control. On the contrary, soluble sugar and Pro content increased by 4- and 16-fold at 175 mM NaCl-induced salt stress. Under 100 mM Na_2_SO_4_-induced salt stress, shoot and root length, leaf area, and root and shoot DW decreased by 17, 28, 18, 30 and 26%, respectively, corresponding to the control [24]. Photosynthetic pigment levels (Chl *a*, Chl *b*, and carotenoid) also decreased by 18, 30, and 15%, respectively. They also recorded a reduction in free amino acid content (27%), soluble sugar (22%), glycine betaine (GB, 13%), Pro (55%), and proteins (5%) at 100 mM Na_2_SO_4_-induced salt stress. In case of secondary metabolites, phenolic compound levels increased by 6%, and tannin content decreased by 1.3%. However, no sharp changes were observed in the case of saponin content at 100 mM Na_2_SO_4_-induced salt stress.

#### 2.1.2. Drought

Water is one of the most important factors for crop growth and productivity in the absence of which morphological and physiological processes and the yield development of plants reduced drastically. In a water deficit condition, about 40–60% crop yield can be reduced [43].

To find out the most critical stage of *C. olitorius* plants regarding water susceptibility, Ayodele and Fawusi [44] imposed drought stress (−6.0 bar) on plants at four different physiological stages (mid-vegetative, flowering, pod formation, and pod filling). Though moisture deficit stress decreased growth and yield in every stage of the plants, because of the highest yield reduction (64%), they marked the flowering stage as the most critical stage, followed by the pod filling stage (47%). Shiwachi et al. [25] exposed two varieties of *C. olitorious* (Yaya and Moroheiya) at three different soil moisture conditions (FC, >75%; light moisture stress, 60–50%; and acute moisture stress, 40–30%) and found that in both varieties plant height reduced in acute and light moisture stress conditions compared to the FC. Plant height reduction in cv. Moroheiya at light and acute moisture stress conditions was recorded as 12% and 23%, respectively, and in cv. Yaya it was 19% and 26%, respectively, compared to the field capacity water level. In both varieties, the highest reduction in leaf number, branches, and leaf area were recorded in the acute moisture stress condition. Similar results were also found by Ewetola and Fasanmi [27] in *C. olitorius* plants where at four different moisture levels (100, 75, 50, and 25% FC), they recorded the highest plant height, stem girth, and leaf area at 75% FC and the lowest at 25% FC, corresponding to the control. At 75% FC, a five-fold increase in biomass yield was observed compared to the control. Yakoub et al. [28] also exposed *C. olitorius* plant at three different water regimes (100, 60, and 40% FC) where the highest reduction in plant height, stem diameter, and leaf area (reduced by 52, 41, and 67%, respectively) were observed at 40% FC. They also observed a reduction in Chl *a*, Chl *b*, and RWC content with the intensity of moisture stress. To cope with the moisture deficit stress, the plant increased the amount of osmolytes, due to which Pro and soluble sugar content increased by eight- and four-fold at 40% FC, respectively, compared to the control. Similar trends were also observed when *C. olitorius* plants were exposed to three different water regimes (30, 60, and 100% ET_c_) where plant height and leaf number sharply increased from the 30% crop water requirement ET_c_ to 60 and 100% ET_c_ [29]. They also stated that because of the reduction in the water regime the drought condition imposed on plants reduced the Chl content index and the yield of the plant. Fasinmirin and Olyufao [26] also grew *C. olitorius* plants in drought conditions by maintaining three irrigation levels (100, 75, and 50% pan evaporation (EP)) and observed reductions in plant height, leaf area, and leaf number plant^−1^ by 7, 10, and 6%, respectively, at 75% EP and 40, 25, and 30%, respectively, at 50% EP over the control. They also recorded 19 and 50% reductions in biomass yield at 75 and 50% EP, respectively, over 100% EP. At a constant drought (8–10% soil moisture) condition, *C. capsularis* plants (cv. CVL-1) failed to complete their life cycle, survived up to 90 d only, and reduced plant height and base diameter by 50 and 42%, respectively, over the control [9,10]. Yumnam et al. [7] exposed *C. olitorius* seedlings in PEG-6000-induced simulated drought stress (−2.0, −3.0, and −4.0 bar) and found maximum reduction in seedling vigor, shoot length, shoot FW, root length, and FW at severe drought stress (−4.0 bar). However, the responses were greatly varied in different genotypes [7].

#### 2.1.3. Waterlogging

Waterlogging is considered one of the main constraints for sustainable agriculture. Waterlogging creates hypoxia in soils, which leads plants towards growth reduction, leaf senescence, reduction in leaf area, stomatal closure etc. [45]. Because of the generation of ROS, plants face oxidative damage at waterlogged conditions. ROS also damages membrane integrity, minimizes the efficiency of PS-II, and causes a significant decrease in P*_n_* [46]. Waterlogging may reduce yields by up to 50% in different crops [47].

Prodhan et al. [9] imposed different levels of standing water on four genotypes of *C. capsularis* (cvs. D-154 and CVL-1) and *C. olitorious* (cvs. O-4 and R-26) and found a severe reduction in plant height by 45, 39, 59, and 61% in CVL-1, D-154, O-4, and R-26 genotypes, respectively, when a 5-cm standing water level was imposed on 30-d-old crop. However, when a 30-cm standing water level was imposed at the mature stage (90-d-old), plant height reduction was much less (3, 3, 9, and 12%) than the previous one. In another experiment, Prodhan et al. [10] demonstrated a similar trend of reduction in stem base diameter at different water regimes, which varied in different genotypes. When the 30-d-old plants were exposed to 5 cm of standing water stem diameters in D-154, CVL-1, O-4, and R-26 genotypes were decreased by 37, 30, 40, and 36%, respectively. However, at the mature stage, this reduction was 0.6, 3, 9, and 8% even at the 30-cm standing water level. This indicates the waterlogging tolerance of jute at mature stages. In another study, Changdee et al. [31] grew *C. olitorius* plants in five different durations of waterlogging (45, 60, 75, 90, and 105 d) and observed a reduction in growth and yield parameters with the increase in waterlogging duration. At 105 d of waterlogging, severe reductions in plant height (53%), stem diameter (36%), leaf area (73%), tap root length (71%), and yield (75%) were recorded over the control. However, adventitious root formation was increased with the increased duration of waterlogging.

Changdee et al. [48] observed aerenchyma cell formation in waterlogged *C. olitorius* plants, which is considered as the key survival factor of plant under waterlogged condition. They also noticed a Casparian band and lignified cell wall in root cells, which prevents root rupture and oxygen loses from aerenchyma. This study is considered as the first report regarding anatomical changes in *C. olitorius* root at waterlogged conditions. Parvin et al. [32] also observed aerenchyma formation in *C. capsularis* plants at a waterlogged condition (2 cm of standing water). They also noticed a decrease in the size of epidermal cells and an increase in the size of pith.

#### 2.1.4. Metal/Metalloid Toxicity

The Earth’s crust contains varying levels of different metals/metalloids. Among these, some are essential for plant cells, viz., Cu, iron (Fe), manganese (Mn), nickel (Ni), and Zn at supra-optimum concentrations, whereas some are harmful, viz., silver (Ag), aluminum (Al), As, Cd, chromium (Cr), cesium (Cs), mercury (Hg), led (Pb), strontium (Sr), and uranium (U), even at low concentrations [49]. Toxic metals/metalloids negatively affect plant morphophysiology and metabolism and finally decrease yield [50]. Plants try to cope with metal/metalloid stress through different defense strategies, viz., exclusion, compartmentalization, complexation, chelation, etc. [51]. Because of the effective defense mechanism and hyperaccumulation of heavy metals, jute is considered as a good candidate for phytoremediation in metal-contaminated soil [52,53]. However, it becomes susceptible at higher concentrations of toxic metals to reduced plant growth, fiber yield, and quality [34,38].

**Figure 2:**
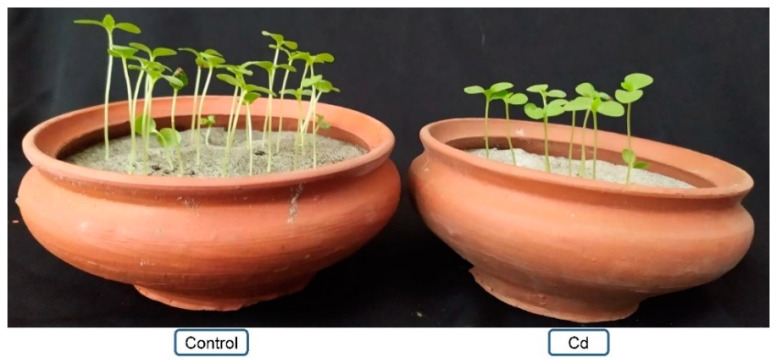
Morphological changes of *C. olitorius* cv. O-9897 seedlings exposed in 0.5 mM CdCl_2_ for 15 days (Cd).

Nizam et al. [33] exposed *C. capsularis* plants at different Cd levels (1, 5, and 10 mg L^−1^) and observed zero emergence of seeds at 10 mg L^−1^ Cd. At 5 mg L^−1^ Cd, survivability % and shoot and root length reduced by 50, 92, and 25%, respectively, in cv. CVE-3 and 35, 73, and 41%, respectively, in cv. BJC-7370 corresponding to the control. Similar results were also recorded by Hassan et al. [34] where *C. olitorius* plants were exposed to different levels of Cd (1, 5, 10, and 20 mg L^−1^). In our recent study, we sowed *C. olitorius* seed in Cd-contaminated (0.5 mM CdCl_2_) growth medium and recorded a reduction in shoot length and survivability in Cd exposure seedlings at 15 days after sowing compared to the control (Figure 2).

In another experiment, Nizam et al. [54] observed a decreasing trend in growth parameters of cvs. BJC-7370 and CVE-3, exposed in Pb-contaminated soil. Islam et al. [55] found that, in *C. olitorius* plants, only higher doses of Cr (200–400 mg kg^−1^ soil) reduced crop growth, and no negative effect was observed at a low level of Cr (100 mg kg^−1^ soil). However, both low and high doses of As (200 and 400 mg kg^−1^ soil) were negatively correlated with growth parameters of *C. olitorius* plants. A combination of a low level of Cr and As (50 mg kg^−1^) slightly alleviated the crop growth and photosynthetic pigments. However, at a high dose, it became toxic to the plant. They also stated that As is more corrosive compared to Cr for *C. olitorius* plants. The germination percentage of *C. capsularis* (cv. Da An Qing Pi) reduced at different levels of Cu (2, 5, 10, 30, and 50 µmol L^−1^) where the maximum reduction (50%) occurred at 50 µmol L^−1^ Cu. Seedling height, FW, and DW were also reduced by 52, 36, and 63%, respectively, over the control [36]. Similar results were also found by Saleem et al. [37] in *C. capsularis* plant at 50 µmol L^−1^ Cu. In another study, Saleem et al. [56] demonstrated that with highly Cu-contaminated soil (natural soil and Cu-contaminated soil ratio 0:1), plant height, shoot FW, shoot DW, and the total Chl content of *C. capsularis* was reduced by 23, 43, 41, and 45%, respectively, over the control. Photosynthesis pigments and chloroplast ultrastructure were also altered by Cu stress and reduced g*_s_*, P*_n_*, C*_i_*, and T*_r_* by 88, 56, 13, and 59%, respectively, corresponding to the control. Parveen et al. [39] also observed a sharp reduction in T*_r_*, P*_n_*, g*_s_*, and C*_i_* at 50 μM (40, 41, 46 and 13%) and 100 μM (58, 67, 77 and 20%) Cu, respectively over the control.

#### 2.1.5. High Light

Light plays a key role in the plant life cycle. As jute is light-sensitive, it showed different responses at a different wave lengths of light. Saleem et al. [2] demonstrated that different red lights (red, dark red, and mixed dark red light) increased plant height, root length, and root/shoot FW and DW compared to white light (control), but blue light and orange light showed negative correlations with these parameters. However, blue and orange light increased the stem diameter by 86 and 84%, respectively, and the root diameter by 83 and 84%, respectively, over the control. They also noticed an increase in total Chl content by 74, 76, and 79% and carotenoid content by 67, 70, and 61% at red, dark red, and mixed dark red light, respectively, corresponding to the control. 

### 2.2. Oxidative Stress and Antioxidant Defense of Jute under Different Abiotic Stresses

One of the obvious consequences of abiotic stress is the excess generation of ROS, which causes oxidative stress. In many plant studies it was observed that ROS metabolism was significantly hampered because of various abiotic stresses (Table 1). Ma et al. [17] tested *C. capsularis* (Huang No.1 and 9511) and *C. olitorius* (Mengyuan and 07–21) genotypes under two different levels of salt (70 and 140 mM NaCl) and observed increased lipid peroxidation (MDA content). At 100 mM Na_2_SO_4_-induced salt stress, MDA content was increased by 110%, which indicated the oxidative stress in plants [24]. It also decreased the activity of GR and increased the activities of CAT, GST, and SOD. At 100 mM Na_2_SO_4_-induced salt stress, oxidized glutathione (GSSG) and glutathione (GSH) were decreased but ascorbate (AsA) content was increased compared to the control.

At 4 d of the water deficit condition, H_2_O_2_ content increased by 142 and 236% in *C. capsularis* and *C. olitorius*, respectively [57]. Plants also showed a sharp increase in MDA content, which indicates the membrane instability in the plant cell. The activities of SOD and CAT also decreased in the water deficit condition. In another experiment, similar results were also recorded by Chowdhury and Choudhuri [58] in *C. capsularis* and *C. olitorius* plants at 2 d of the water deficit condition.

In *C. olitorius* plants, Islam et al. [55] observed a sharp increase in MDA content due to As stress compared to Cr stress. However, a combination of low levels of As and Cr stress (50 mg kg^−1^) decreased the MDA content, which indicated less oxidative damage. Saleem et al. [37] also reported that the maximum content of MDA and H_2_O_2_ and the activities of SOD, POD, CAT, and APX were observed at the Shang Huo Ma genotype exposed in Cu-contaminated (50 µmol L^−1^) media. Similarly, with the increasing levels of Cu (50 and 100 μM), MDA content increased by 108 and 228%, respectively, in *C. capsularis* over the control [39]. In another experiment, Saleem et al. [59] observed a higher MDA content in *C. capsularis* at 100 μM Cu (CuSO_4_.5H_2_O), which also increased the activities of POD and SOD.

Oxidative stress was induced by blue light and orange light in *C. capsularis* plants and increased MDA (83 and 90%, respectively) content compared to the control [2]. They also increased the activities of SOD (86 and 84%) and POD (83 and 82%) in stressed plants.

## 3. Jute Quality under Abiotic Stress

The quality of jute fiber has great importance for industrial use, which could be deteriorated under different abiotic stress conditions like metals/metalloids, waterlogging, salinity, etc. Though several articles have been published related to morphological, physiological, and biochemical responses of jute, only a few reports have discussed the fiber quality under stressed conditions [30,60,61]. Abiotic stress is responsible for the reduction in reed length, fiber color, the fiber elongation rate, and fiber breaking strength, and ultimately it reduces the marketability of the fibers [6,8].

Ghorai et al. [60] imposed waterlogging stress on three cultivars of jute (*C. olitorius* cv. JRO-524; *C. capsularis* cvs. Hybrid-C and JRC-212) and found that reed length (length of fiber) was reduced by 19–24% under different water heads (0, 4–5, 8–10, 10–15, 16–20, 18–25, and 22–30 cm) compared to the control (40 cm raised bed). Along with this, the basal region of jute plants become hard and dark-colored because of the waterlogging condition, which caused a reduction in fiber quality.

Key determinants of fiber quality parameters, e.g., reed length, fiber strength, fiber color, and the number of fiber layers in a fiber strand, were adversely affected by the waterlogging stress of *C. olitorius* (cv. JRO-524). Both reed length and fiber strength were reduced by 11−43% and 12−55%, respectively, under different water heads (0, 5, 10, 15, 20, 25, and 30 cm) compared to the control (well-drained condition). Under waterlogging, fiber layers become thinner in the fiber strand, which ultimately affects the fiber strength. A higher reduction of fiber layers per fiber strand was observed at the 30 cm waterlogged condition compared to the control. Furthermore, because of waterlogging stress, hard barky bottoms were observed, which also deteriorated the quality of fibers [30].

Under water deficit stress (WDS), fiber strength was significantly higher in tolerant genotypes of *C. olitorius* (OIN 631, OIN 632, and OIN 633) compared to the control. In contrast, a reduction of fiber strength was observed in susceptible genotypes (OIN 694, OIN 873, and OIN 875) under WDS. It was also noticed that fiber fineness was reduced in tolerant genotypes, but, in cases of susceptible genotypes, it was increased prominently when it was subjected to the water deficit condition [8].

Upon exposure to salinity stress (0, 100, 150, and 250 mM NaCl), K^+^ was decreased significantly in the leaves and shoots of all tested cultivars of *C. capsularis* (JRC-698, JBC-5, JRC-532, JRC-321, and JRC-517) compared to the control. Decreasing K^+^ ion in plants reduced the fiber quality, as K content is considered as a vital quality element for fiber [6].

Saleem et al. [61] conducted an experiment with two varieties of *C. capsularis* (cvs. Hong Tie Gu Xuan and Gu Ba Chang Jia) that were cultivated in highly Cu-contaminated soil (2221 mg kg^−1^) and incorporated with different doses of P, viz., 30, 60, and 120 kg ha^−1^, which significantly affected both fiber yield and quality. Fiber quality parameters like fiber diameter, fiber breaking strength, and the fiber elongation rate, were reduced under highly Cu-contaminated soil. When P was applied at the rate of 60 kg ha^−1^, fiber diameter (43 μM), fiber breaking strength (3.9 cN), and the fiber elongation rate (40%) were highest in cv. Hong Tie Gu Xuan. However, quality parameters were reduced at 120 kg ha^−1^ P.

## 4. Contrasting Abiotic Stress Responses of *C. capsularis* and *C. olitorius*

*C. capsularis* and *C. olitorius* are two commercial species of jute, which act differently at different abiotic stresses. They showed different tolerance mechanisms when exposed to abiotic stresses.

Chaudhury and Choudhuri [62] exposed *C. capsularis* (JRC-212) and *C. olitorius* (JRO-632) at 200 mM NaCl-induced salt stress where RWC and P*_n_*, decreased by 6 and 83% in JRC-212 and 23 and 95% in JRO-632. Higher accumulation of Na^+^ and Cl^−^ were recorded at *C. olitorius*, which indicates the salt susceptibility of *C. olitorius* plants. Ma et al. [17] observed higher MDA content in Mengyuan and 07–21 (*C. olitorius*) over Huang No.1 and 9511 (*C. capsularis*) genotypes. The activities of CAT and SOD also decreased in Mengyuan and 07–21, which indicated the higher salt tolerance of *C. capsularis* over *C. olitorius*.

Prodhan et al. [9] demonstrated that when 30-d-old seedlings were exposed at constant drought condition (8–10% moisture) *C. capsularis* cv. CVL-1 survived only 90 d but *C. olitorius* cv. O-4 could survive up to 120 d and complete their life cycle. Reduction in plant height was more severe in CVL-1 (50%) compared to O-4 (38%) at 90-d-old seedlings. A similar trend was also observed in the case of stem diameter of both varieties [10].

Prodhan et al. [9] exposed four genotypes of *C. capsularis* (cv. D-154 and cv. CVL-1) and *C. olitorius* (cvs. O-4 and R-26) at a 5-cm standing water level and observed severe reduction of plant height in *C. olitorius* (59 and 61%) compared to *C. capsularis* (45 and 39%). The stem diameter of *C. olitorius* was greatly reduced at different water levels compared to *C. capsularis.*

Several studies [39,52,56,63] have proven that both species of jute have good phytoremediation capacity and can mitigate metal/metalloid toxicity. However, in the case of Cd stress, *C. olitorius* plants were able to establish at 1, 5, 10, and 20 mg L^−1^ Cd [63], but *C. capsularis* (cvs. BJSC-7370 and CVE-3) failed to germinate at 10 mg L^−1^ Cd [34], which proves the susceptibility of *C. capsularis* to Cd stress.

**Figure 3:**
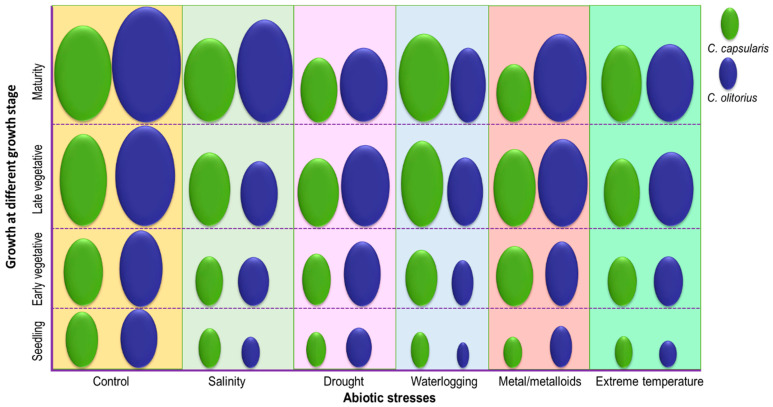
Hypothetical comparison matrix of the relative tolerance of *C. capsularis* and *C. olitorius* to major abiotic stresses. This a general trend based on the various published literature and preliminary studies. The actual tolerance varies with the stress levels and genotypes.

We have illustrated the relative tolerance of *C. capsularis* and *C. olitorius* to major abiotic stress based on the known literature and our preliminary studies (Figure 3). As fewer studies have been found regarding the specific stress tolerance of these two species, it is hard to specify the comparison among two jute species. Further studies should be conducted to specify the different stress tolerance species of jute. This can be a promising phenomenon to create a further scope of the investigation at the molecular level to tailor stress tolerance parameters of *C. capsularis* and *C. olitorius*.

## 5. Approaches in Enhancing Abiotic Stress Tolerance in Jute

Environmental stress greatly hampered jute plant growth and physiology. Implementation of different agronomic management practices through the application of different kinds of nutrients, phytohormones, nano-particles, plant extracts, chelates, etc. improved plant growth conditions (Table 2) and can be a helpful tool to alleviate the negative effect of abiotic stress.

### 5.1. Fertilizer Management

Plant nutrients play a vital role in many morphological and physiological processes in the plant. They also help to meet up high crop yield even at different stress conditions. The requisite amount of fertilizer helps to mitigate stress, though the excess amount may induce toxicity in plants. Fertilizer management can be a promising phenomenon in introducing abiotic stress tolerance in jute. The application of different doses of P (30, 60, and 120 kg ha^−1^ P) in Cu-contaminated soil improved the growth condition and reduced oxidative stress in *C. capsularis*, which ultimately improved yield and yield quality [61].

### 5.2. Early Sowing

By altering the sowing time of the crop, farmers can avoid stress conditions in some cases. Though jute is waterlogging tolerant at the mature stage, at the vegetative stage they cannot withstand waterlogging up to 30-d-old [67]. Early sowing can be an effective approach to avoid waterlogging caused by heavy rainfall.

### 5.3. Application of Phytohormones

Phytohormones are termed as a chemical messenger, which plays a crucial role in regulating the growth, physiology, and biochemical responses of plants at abiotic stress [68]. Although abscisic acid (ABA) inhibited plant growth at a higher concentration, at a lower concentration (0.01 mM), foliar application of ABA helped to mitigate drought stress in both *C. capsularis* and *C. olitorius* [58]. Another phytohormone, viz., KN can be used to mitigate the effect of NaCl-induced salt stress in both *C. capsularis* and *C. olitorius* species [62]. Metal uptake is also reduced by phytohormones, viz., GA in Cu-contaminated soil and improved growth parameters and reduced oxidative damage in plants [66].

### 5.4. Seed Priming

Plant growth and productivity are negatively hampered by abiotic stress, which can be alleviated by seed priming. Pretreatment of seeds with calcium chloride (CaCl_2_) increased structural integrity of cell wall and improved antioxidant defense of *C. capsularis* at the water deficit condition [65]. Seed priming with 10% parsley extracts helps to improve morpho-physiological responses of *C. olitorius* at Na_2_SO_4_-induced salt stress [24].

### 5.5. Stress-Tolerant Varieties

Yield losses due to abiotic stress can be effectively reduced by introducing stress-tolerant varieties. In Bangladesh, the Bangladesh Jute Research Institute (BJRI) had released salt-tolerant cvs. BJRI Deshi Pat-8 and BJRI Deshi Pat-10 are moderately salinity-tolerant and can be introduced in saline-prone areas of the southern coastal districts of Bangladesh.

## 6. Genetic Approaches in Enhancing Jute Tolerance to Abiotic Stress

Enhancement of stress tolerance in jute through the conventional breeding program is greatly challenging because of the incomplete and narrow gene pool of jute. In jute-producing countries, a conventional breeding program was followed to develop jute variety for higher fiber yields. To produce quality jute fiber under different forms of abiotic stress, improved transgenic jute plants should be developed by modifying the jute genome sequence through approaching convenient techniques.

With the increased study of the salt-stress tolerance of jute, it has become a potential candidate for growing under saline soil. According to Islam et al. [69], ROS-scavenging enzymes were up-regulated under saline conditions (150 mM NaCl) in jute plant (*C. olitorius* cv. O-72) through transfer DNA (T-DNA) technology using *Escherichia coli* (K-12) *katE* gene, which pulled the transgenic plant to the reproductive stage by improving the salt tolerance capacity over the control. This might be a feasible option to develop the breeding program. In another experiment, Ma et al. [70] reported that through expressing a gene encoding a stress-responsive protein complex, jute seedlings were adapted to salt stress (160 mM NaCl, 4 d) by changing signal transduction, reducing oxidative stress, conserving energy, enhancing cellular metabolism, and altering root structure.

To explore the possible salt-stress-responsible gene, Yang et al. [71] reported that, along with different transcription factors, a smaller amount of differentially expressed unigenes (DEGs) were implicated in different metabolic traits that enhanced stress tolerance in jute, such as the ABA signaling pathway, plant hormone signal transduction, and metabolism of cystine/methionine, and these were mainly noticeable in the root tissue of salt-tolerant genotypes. Similarly, another three relevant pathways enriched in root, namely, Ca^2+^- and mitogen-activated protein kinase signaling pathways and oxidative phosphorylation pathways, were identified by Yang et al. [72], which were involved in the mechanism of salt stress tolerance in two jute species (*C. capsularis* and *C. olitorius*).

Yang et al. [73] subsequently constructed a high-throughput quantitative trait locus (QTL) mapping, which consisted of 4839 markers on seven linkage groups (LG) that ranged from LG1–7 for improving salt-stress tolerance at the germination stage. Among these, three highly and thirteen slightly effective QTLs were found on four LGs under two salt stress treatments (140 and 160 mM NaCl), which were mainly implicated in salt tolerance. The major QTL, qJST-1, was identified under both salt stress treatments, which was observed with the highest phenotypic variation in germinated seeds (12 and 20%). This approach can be used in improving germination under salt stress in jute.

Sawarkar et al. [74] studied sixty *C. olitorius* genotypes to isolate drought-tolerant as well as maximum-fiber-yielding lines and screened out fifteen lines with promoted early maturity and increased plant height, stem diameter, fiber weight, and thickness. Seed pre-treatment with several primers, such as CaCl_2_, CaNP, and β- aminobutyric acid, also improved the drought stress tolerance through expressing transcripts of jute (JEC 1–18), which were involved in the up-regulation of Pro, CAT, and POD and the down-regulation of Pro oxidase [65]. Yang et al. [75] conducted a comparative study between *C. olitorius* (drought-tolerant) and *C. capsularis* (drought-sensitive) species and observed that tolerant species were enriched with higher DEGs compared to sensitive ones. Yang et al. [75] also introduced the contribution of the two most important pathways in drought-stress tolerance in *C. olitorius* and *C. capsularis*, viz., phenylpropanoid biosynthesis and the peroxisome pathway. Several TFs, namely, no apical meristem (NAM), *Arabidopsis* transcription activation factor, and cup-shaped cotyledon 2 occupied the *C. capsularis NAC1* (*CcNAC1*) gene and were expressed under drought stress (20% PEG) to mitigate the negative effect by speeding the early maturity of *C. capsularis* [76]. Additionally, Zhang et al. [77] found that regulation of *NAM-2-like* genes can be significantly involved in drought tolerance in *C. capsularis*.

Niu et al. [78] evaluated the expression of ubiquitin-conjugating enzyme (*UBC*) and chaperon protein under PEG-induced (20%) drought stress, while, upon exposure to salt stress (200 mM NaCl), an Ras-related small GTP-binding nuclear protein was detected as the most stable candidate gene to study the survival capacity of jute under these stresses. Along with *UBC*, elongation factor alpha (*EF1*α) can be used as a reference gene to mitigate low temperature stress in *C. olitorius* [79].

Additionally, the positive involvement of Ca-dependent protein kinase (CDPK) was established by Ahmed et al. [80], and they found the contribution of *CoCDPK6*, *11*, and *12* genes (in *C. olitorius*) and *CcCDPK8*, *10*, and *18* genes (in *C. capsularis*) in both salt and drought stress tolerance and quality improvement in fiber yield.

To develop transgenic jute plants, RNA interference technology can also be adopted. To investigate the participation of microRNA (*miRNAs*) and the related target genes to metal stress tolerance, Hauqe et al. [81] found that up-regulation of *miR319* and a gene ATP-binding cassette (ABC) transport played a fundamental role as a metal transporter and increased As (250 µM NaAsO_2_) stress tolerance in jute (*C. olitorius* cv. O-9897). In addition, Hauqe et al. [81] reported jute as a metal accumulator because of the down-regulation of two *miRNAs*, i.e., *miR159* and *miR167*, and their marked genes ABC and auxin responsive factor 8 (ARF8) in response to Mn (25 mM KMnO_4_) and Cr (25 mM K_2_Cr_2_O_7_), and they improved the remedial capacity of *C. olitorius* against Mn and Cr.

By following the particle bombardment method, Bhattacharyya et al. [82] transferred a bialaphos-tolerant gene into *C. capsularis* cv. JRC-321 to obtain a herbicide-tolerant transgenic jute plant. It might be a great opportunity to introduce stress-related targeted genes into the *Corchorus* species by in vitro approaches.

In Bangladesh, recently whole genome sequences of both the cultivated species, viz., *C. capsularis* and *C. olitorius*, were discovered, which might facilitate the progress of improved breeding technologies [16]. Stress-tolerant jute cultivars can be invented by increasing the studies at the gene level under different abiotic stresses. Thus, transgenic approaches might be a successful measure to increase quality fiber production and improve abiotic stress tolerance in jute.

## 7. Conclusions

Understanding plant abiotic stress responses of crop plants is a complex task as the plant acts differently under various abiotic stresses, and it also depends on the levels and duration of the stresses. Unlike many other crops, the response of jute to abiotic stress is very complex, and such responses are dependent on the plant’s age, species, and genotype. Therefore, the stress tolerance mechanism of jute is multidimensional, and understanding the gene regulation, signaling cascade, and adaptive mechanism is an important task for plant physiologists, breeders, and molecular biologists. In some Asian countries like Bangladesh, jute is the main fiber crop and is often called the “golden fiber.” Therefore, revealing the natural secret of jute and exploiting its genetic basis would result in suitable jute varieties tolerant to abiotic stress. Although there are some tolerant varieties developed by researchers, these are not sufficiently tolerant. Researchers have unrevealed the jute genome in the last decade, and there is scope for further molecular advancement towards tolerant jute varieties. Approaches in enhancing the antioxidant defense in plants would be another area of research that can mitigate oxidative stress in jute plants. Manipulating agronomic practices can also contribute to adapting the abiotic stresses. Therefore, integrating plant physiology, agronomy, and plant breeding should be practiced to gain sustainable development in jute production in the era of climate change. Unfortunately, there is still lack of studies regarding jute responses and tolerance to extreme temperature, which would be a topic of further research.

## Figures and Tables

**Table 1:** Jute (*C. capsularis* and *C. olitorius*) responses to different abiotic stresses.

Species	Stress Level	Effects	References
**Salt stress**			
*C. olitorius*, *C. capsularis*	140 mM NaCl	Increased malondialdehyde (MDA) content and peroxidase (POD) activity. Inhibited catalase (CAT) and superoxide dismutase (SOD) activities.	[17]
*C. olitorius*	4 and 6 ds m^−1^ NaCl	Reduced plant height, number of leaves plant^−1^, root/shoot length, and dry weight (DW). Decreased stem diameter and fiber yield.	[18]
*C. olitorius* (cv. O-9897)	200 mM NaCl	Reduced shoot length (59%), root length (60%), relative water content (RWC; 21%), and the SPAD value (13%).	[19]
*C. olitorius*	160 mM NaCl	Reduced germination, shoot and root length, fresh weight (FW), and DW.	[20]
*C. capsularis* (cv. CVL-1)	100 mM NaCl	Decreased germination (75%), plant height (74%), number of leaves (65%), and DW (73%).	[21]
*C. capsularis*	200 mM NaCl	Inhibited germination, plant height and branches plant^−1^, number of seed capsule^−1^, seed yield plant^−1^, and 1000-seed weight. Increased false seed content.	[22]
*C. capsularis* (cv. JRC-517)	250 mM NaCl	Reduced shoot length (50%), root length (40%), number of leaves (>70%), and RWC (39%). Decreased K^+^, chlorophyll (Chl) *a* and Chl *b* content Increased Na^+^ content.	[6]
*C. olitorius*	175 mM NaCl	Reduced germination (60%), number of ramifications (57%), leaf area (69%), FW (40%), number of pods plant^−1^ (49%), and number of seeds pod^−1^ (37%). Decreased net photosynthesis (P*_n_*), stomatal conductance (g*_s_*), and transpiration rate (T*_r_*) by 75, 86, and 75%, respectively. Increased Pro and soluble sugars by 16- and 4-fold.	[23]
*C. olitorius*	100 mM Na_2_SO_4_	Reduced shoot and root length, leaf area, and root and shoot DW by 17, 28, 18, 30, and 26%, respectively. Decreased Chl *a*, Chl *b*, and carotenoid content by 18, 30, and 15%, respectively. Reduced free amino acids (27%), soluble sugar (22%), proteins (5%), and tannin content (1.3%). Increased phenolic compounds by 6%. Increased MDA (110%) and proline (Pro) (55%). Inhibited the activities of CAT, glutathione-*S*-transferase (GST), and SOD. Reduced glutathione reductase (GR) activity.	[24]
**Drought stress**			
*C. capsularis*, *C. olitorius*	8–10% soil moisture	Reduced plant height (35–50%).	[9]
*C. capsularis*, *C. olitorius*	8–10% soil moisture	Decreased base diameter (16–42%).	[10]
*C. olitorius* (cvs. Yaya, Moroheiya)	Acute moisture stress (40–30%), light moisture stress (60–50%)	Inhibited plant height, number of nodes on stem, and node length. Decreased leaf area, root DW, and fiber yield.	[25]
*C. olitorius*	50% pan evaporation (EP)	Reduced plant height (40%), leaf number plant^−1^ (30%), leaf area (25%), and yield (50%).	[26]
*C. olitorius*	25% field capacity (FC)	Decreased plant height, leaf area, leaf number plant^−1^, and stem girth. Reduced yield by 80%.	[27]
*C. olitorius*	40% FC	Reduced plant height (52%), stem diameter (41%), and leaf area (67%). Increased Pro and soluble sugar content by 8- and 4-fold.	[28]
*C. olitorius*	Polyethylene glycol (PEG-6000) (−2.0, −3.0, and −4.0 bar)	Decreased shoot and root length, FW, and DW.	[7]
*C. olitorius* (cvs. OIN 694, OIN 873, OIN 875)	Water deficit, 10 d	Decreased plant height, root length, and stem diameter. Decreased RWC, photosynthetic carbon assimilation, P*_n_*, and T*_r_.* Deteriorated fiber strength and fineness. Increased Pro and flavonoid contents. Reduced polyphenols contents.	[8]
*C. olitorius*	30% crop water requirement (ET_c_)	Reduced plant height (23%) and leaf number (34%). Decreased Chl content index and yield.	[29]
**Waterlogging stress**			
*C. capsularis*, *C. olitorius*	5 cm standing water imposed on 30-d-old seedlings	Reduced plant height (39–61%).	[9]
*C. capsularis*, *C. olitorius*	5 cm standing water imposed on 30-d-old seedlings	Decreased base diameter (30–40%).	[10]
*C. olitorius*	5, 10, 15, 20, 25, and 30 cm standing water	Reduced plant height, tap root DW, and basal diameter. Decreased stomatal resistance, P*_n_*, and T*_r_.* Decreased fiber yield by 20–60%. Inhibited fiber length (11–43%) and fiber strength (12–55%).	[30]
*C. olitorius*	Waterlogging, 105 d	Decreased plant height (53%), shoot DW (87%), stem diameter (36%), leaf area (73%), tap root length (71%), and yield (75%). Increased adventitious root formation. Developed aerenchyma tissue in adventitious root.	[31]
*C. capsularis*	2 cm standing water	Induced aerenchyma formation. Increased pith size. Reduced xylem vessels. Decreased epidermal cell size.	[32]
**Metal/metalloid stress**			
*C. capsularis* (cv. CVE-3)	5 mg L^−1^ cadmium (Cd)	Decreased survivability (92%), shoot length (92%), and root length (25%). Reduced shoot and root DW.	[33]
*C. olitorius*	1, 5, 10, and 20 mg L^−1^ Cd	Reduced root and shoot FW. Alleviated Pro content.	[34]
*C. capsularis* (cv. BJC-7370)	98.25 mg kg^−1^ arsenic (As)	Reduced germination (19%), survivability (9%), and stem girth (53%). Inhibited plant height and dry biomass production.	[35]
*C. capsularis* (cv. Da An Qing Pi)	50 µM copper (Cu) (CuSO_4_.5H_2_O)	Inhibited germination (50%), plant height (52%), shoot. FW (36%), and DW (63%). Increased POD (46%) and SOD (29%) activities.	[36]
*C. capsularis* (cv. Shang Huo Ma)	50 µM Cu (CuSO_4_.5H_2_O)	Reduced germination, plant height, total Chl content, shoot FW, and DW. Increased MDA and H_2_O_2_ content. Decreased CAT and ascorbate peroxidase (APX) activities.	[37]
*C. capsularis*	100 µM Cu (CuSO_4_.5H_2_O)	Decreased plant height (52%), FW (22%), DW (35%), and stem diameter (25%). Inhibited, P*_n_*, T*_r_*, intercellular CO_2_ (C*_i_*), and g*_s_* content Increased MDA (475%) and Pro content (446%). Increased POD and SOD activities.	[38]
*C. capsularis*	100 µM Cu (CuSO_4_.5H_2_O)	Decreased plant height (37%), FW (20%), DW (35%), and stem diameter (33%). Reduced T*_r_*, P*_n_*, g*_s_*, and C*_i_* by 58, 67, 77, and 20%, respectively. Increased MDA (229%) content and SOD (476%) and POD (107%) activities.	[39]

**Table 2:** List of different protectants and their protective roles in enhancing abiotic stress tolerance in jute.

Species	Stress Level	Protectants	Protective Effects	References
*C. olitorius*, *C. capsularis*	160 and 200 mM NaCl	0.09 mM kinetin (KN), 4 mM glutamic acid, 5 mM calcium nitrate	Higher P*_n_*, T*_r_*, and water use efficiency.	[62]
*C. olitorius*	1, 5, 10, and 20 mg L^−1^ Cd	5 mM citric acid (CA)	Higher shoot and root biomass. Reduced Cd uptake.	[34]
*C. capsularis*	Drought stress (24 h)	CaCl_2_ nanoparticle (CaNP), and β- aminobutyric acid (20 µg ml^−1^)	Higher Pro content. Increased CAT and POD activities.	[64]
*C. capsularis*	100 µM Cu (CuSO_4_.5H_2_O)	3 mM ethylenediaminetetra acetic acid (EDTA)	Increased plant height (9%), FW (6%), DW (11%), and stem diameter (7%). Higher P*_n_*, T*_r_*, C*_i_*, and g*_s_* by 8, 13, 14, and 27%. Increased total Chl content by 21%. Reduced MDA and Pro content. Decreased POD and SOD activities.	[38]
*C. capsularis*	Cu (2221 mg kg^−1^ soil)	30, 60, and 120 kg ha^−1^ P	Increased plant height and shoot FW and DW. Reduced MDA and Pro content. Higher fiber yield and improved quality.	[61]
*C. capsularis*	Cu-contaminated soil (2221 mg kg^−1^)	100 mg L^−1^ gibberellic acids (GA)	Increased plant height and shoot FW and DW by 31, 31, and 36%, respectively. Improved P*_n_*, T*_r_*, C*_i_*, and g*_s_.* Reduced MDA (51%), H_2_O_2_ (54%), and electrolyte leakage (EL; 39%). Increased CAT (25%), POD (40%), SOD (54%), and APX (28%) activities.	[65]
*C. capsularis*	80 µM Cu (CuSO_4_.5H_2_O)	3 mM EDTA + 3 mM citric acid (CA)	Increased plant height (55%), stem diameter (24%), and shoot FW (36%) and DW (31%). Higher P*_n_*, T*_r_*, C*_i_*, and g*_s_* by 55, 170, 20, and 175%, respectively. Reduced MDA (37%), H_2_O_2_ (28%), and EL (43%). Increased CAT (63%), POD (68%), SOD (142%), and APX (48%) activities.	[66]
*C. capsularis*	100 µM Cu (CuSO_4_.5H_2_O)	2 mM CA	Increased plant height (40%), stem diameter (18%), and shoot FW (41%) and DW (33%). Increased P*_n_* (50%), T*_r_* (59%), and g*_s_* (20%) content. Reduced MDA (27%) and Pro (11%) content.	[39]
*C. olitorius*	100 mM Na_2_SO_4_	10% parsley extracts (pretreatment)	Increased shoot height, root length, and leaf area by 16, 10, and 19%, respectively. Higher Chl *a* and Chl *b* content. Decreased MDA, Pro, tannins, and GB content.	[24]

## Data Availability

All relevant information is present in this manuscript.
